# Slow response of surface water temperature to fast atmospheric variability reveals mixing heterogeneity in a deep lake

**DOI:** 10.1038/s41598-024-58547-0

**Published:** 2024-04-11

**Authors:** Marina Amadori, Mariano Bresciani, Claudia Giardino, Henk A. Dijkstra

**Affiliations:** 1 Institute for Electromagnetic Sensing of the Environment, National Research Council, 20133 Milan, Italy; 2 Department of Physics, Institute for Marine and Atmospheric Research Utrecht, Utrecht University, 3584 CC Utrecht, The Netherlands; 3 Department of Civil, Environmental and Mechanical Engineering, University of Trento, 38123, Trento, Italy

**Keywords:** Climate sciences, Limnology, Ocean sciences

## Abstract

Slow and long-term variations of sea surface temperature anomalies have been interpreted as a red-noise response of the ocean surface mixed layer to fast and random atmospheric perturbations. How fast the atmospheric noise is damped depends on the mixed layer depth. In this work we apply this theory to determine the relevant spatial and temporal scales of surface layer thermal inertia in lakes. We fit a first order auto-regressive model to the satellite-derived Lake Surface Water Temperature (LSWT) anomalies in Lake Garda, Italy. The fit provides a time scale, from which we determine the mixed layer depth. The obtained result shows a clear spatial pattern resembling the morphological features of the lake, with larger values (7.18± 0.3 m) in the deeper northwestern basin, and smaller values (3.18 ± 0.24 m) in the southern shallower basin. Such variations are confirmed by in-situ measurements in three monitoring points in the lake and connect to the first Empirical Orthogonal Function of satellite-derived LSWT and chlorophyll-a concentration. Evidence from our case study open a new perspective for interpreting lake-atmosphere interactions and confirm that remotely sensed variables, typically associated with properties of the surface layers, also carry information on the relevant spatial and temporal scales of mixed-layer processes.

## Introduction

Interactions between a lake and the overlying atmosphere dominantly occur in the surface mixed layer. Here atmospheric momentum and buoyancy fluxes cause turbulent flows in the lake which are central to the exchange of tracers such as oxygen, affecting trophic conditions. The vertical extent of such a turbulent flow is bounded by the mixed layer depth, here indicated by *h*, which in statistical equilibrium is set by the depth that turbulent eddies can penetrate in the background lake stratification^1^.

Most limnological studies compute this key length scale referring to the seasonal mixed layer^2^. Such estimate is often derived from in-situ temperature profiles with inadequate vertical resolution and/or instrumentation accuracy and with a variety of methodologies often inconsistent among each other^3,4^. In addition to this, an actively mixed layer resides in the upper part of the seasonal epilimnion^5^ and varies spatially depending on the local water depth, atmospheric forcing^6^, and local internal motions^7^. To determine the lake wide variation of *h*, in-situ measurements are not practical as they are usually very localised. Satellite measurements might allow to fill this gap by easily observing the complete lake surface^8^, but they are typically associated with surface properties only, such as lake surface water temperature (LSWT). Despite providing information at the very surface water layer (often identified as a skin layer^9^), surface water temperature (from now on indicated by $T_w$ [^∘^C]) is the only lake physical quantity that air-water energy fluxes depend on.

The vertical extent of *h* largely affects $T_w$ by actively modulating the surface layer thermal inertia^10^. Conceptual models such as air2water^11^ and FLake^12^ build on this assumption by modelling the surface layer through bulk heat budget equations and have been successfully applied at local^13^ to global^14^ scale to address climate-driven changes of lake’s thermal regimes worldwide^15–17^ (see also^18^ for an extensive review on this topic). Further applications of such simplified models on single study sites have also shown their potential to describe the tight relation between $T_w$, *h* and the local lake depth^19^ in warming climate scenarios, with a significant computational effort saving compared to more complex 1D models applied in multi-column mode^20^ as well as 3D^21^ models.

In this paper, we present a new approach to determine estimates of *h* from satellite surface measurements of $T_w$. The idea is based on the work on stochastic climate models^22^, which is applicable when the decorrelation time scale of the atmospheric forcing is much smaller than the equilibration time scale of the lake’s mixed layer temperature. Due to this time scale separation, the atmospheric forcing can be modelled by noise and the thermal response of the lake surface temperature follows a red noise stochastic process. From the statistical properties of this red noise process, the values of the mixed layer depth *h* can then be determined. The theory was successfully applied to explain the red noise characteristics of variability of sea surface temperature (SST) under random buoyancy forcing^23^ and was an important component of the 2021 Nobel Prize work of Hasselmann^24^.

As far as we know, this theory has not been applied to lakes yet, although also a similar separation of time scales occurs. However, research in this direction has been paved by^25,26^, who have shown the potential of correlation timescales of LSWT anomalies for assessing the amplitude of a lake’s response to air temperature anomalies. In the “Methods” section, we present how this theory can be used to determined the relationship between $T_w$ and *h* and what data are needed to accomplish this. As a proof of concept, we apply this theory to the smaller lacustrine environment of a well known case study (Lake Garda, the largest Italian lake). Here, data from remote sensing and in-situ observations are available, as well as knowledge on its main morphological and thermal features. Our aim is investigating whether LSWT variability can be explained as an integral response to atmospheric white noise such that an estimate of mixed layer depth can be derived. Secondly, we aim at demonstrating that such behaviour leaves a signature of the main spatial patterns of remotely sensed water quality parameters, in particular LSWT and chlorophyll-a concentration, revealing underwater heterogeneity and opening a new perspective for Earth Observation-based climate studies concerning lakes. In the discussion we summarise our novel approach and describe its application potential and its limitations.

## Methods

### Theory

Consider a lake surface layer of constant depth *h* which is subject to a random buoyancy forcing in the form of a net surface heat flux $Q_{net}$ [Wm^-2^]. The lake water has a constant water density $\rho _w$ [kgm^-3^] and a constant heat capacity $c_{pw}$ [J kg^-1^^∘^C^-1^]. Assuming that the heat flux at the bottom of the layer is negligible compared to that at the surface, the integration of the heat equation over a layer at a particular location gives

1 $$\begin{aligned} \frac{dT_w}{dt} = \frac{Q_{net}}{\rho _w c_{pw} h}, \end{aligned}$$

where $T_w$ is the layer integrated temperature. If the upper layer is well mixed, this temperature is equal to the LWST. After a linearisation of the surface energy balance of the lake around the background state (which can be a long term mean state), the surface heat flux can be approximated^27^ as $Q_{net} = \alpha (T_a - T_w)$ where $T_a$ is the atmospheric temperature just (for example, 2 m) above the lake and $\alpha$ is an exchange coefficient. In this way, Eq. (1) becomes

2 $$\begin{aligned} \frac{dT_w}{dt} = -\gamma T_w + \gamma T_a, \end{aligned}$$

with

3 $$\begin{aligned} \gamma = \frac{\alpha }{\rho _w c_{pw} h}. \end{aligned}$$

When the atmospheric forcing is deterministic, the solution to Eq. (2) is easily determined (by the variations of constant method) as

4 $$\begin{aligned} T_w(t) = e^{-\gamma t} (T_w(0) + \gamma \int _0^t e^{\gamma s} T_a(s) ds) \end{aligned}$$

In equation (4), $\gamma$ quantifies the exponential decay of $T_w$ as a function of the heat exchanges through the atmosphere, which is inversely proportional to the mixed layer depth. The exponential decay evident from Eq. (4) is embedded in models of increasing complexity and it is, for example, explicitly accounted for in parameter $a_4$ of air2water^11^.

However, in general, $T_a$ is a rapidly varying function of the time and it can be considered as noise on the time scale of the evolution of $T_w$. Hence, one can decompose both $T_w$ and $T_a$ into their long term time mean and a fluctuation on this mean, i.e. $T_w = \bar{T}_w + \tilde{T}_w$ with a similar expression for $T_a$. In case the stochastic properties of $\tilde{T}_a$ are such that it can be modelled as additive white noise, the Eq. (2) turns into an Itô stochastic differential equation

5 $$\begin{aligned} dX_t = -\gamma X_t dt + \sigma dW_t \end{aligned}$$

where $X_t$ is the stochastic process representing $\tilde{T}_w$, $W_t$ is a Wiener process and $\sigma$ represents the amplitude of the noise.

The solution of Eq. (5) is an Ornstein–Uhlenbeck process given by

6 $$\begin{aligned} X_t = e^{-\gamma t} (X_0 + \sigma \int _0^t e^{\gamma s} dW_s ) \end{aligned}$$

where the second term is an Itô stochastic integral. The statistical properties of this process are well known^28^. In statistical equilibrium it has zero mean, a variance given by $\sigma ^2/(2 \gamma )$, an exponentially decaying autocorrelation function with rate $\gamma$, and a Lorentzian spectrum given by

7 $$\begin{aligned} P(f) = \frac{\sigma ^2}{\gamma ^2 + f^2} \end{aligned}$$

with frequencies *f*. The challenge here is whether we can determine $\gamma$, and hence the mixed layer depth *h*, over a period of the year where this depth is reasonably constant, from the statistical properties of observed $\tilde{T}_w$.

### Study area and available data

**Figure 1:**
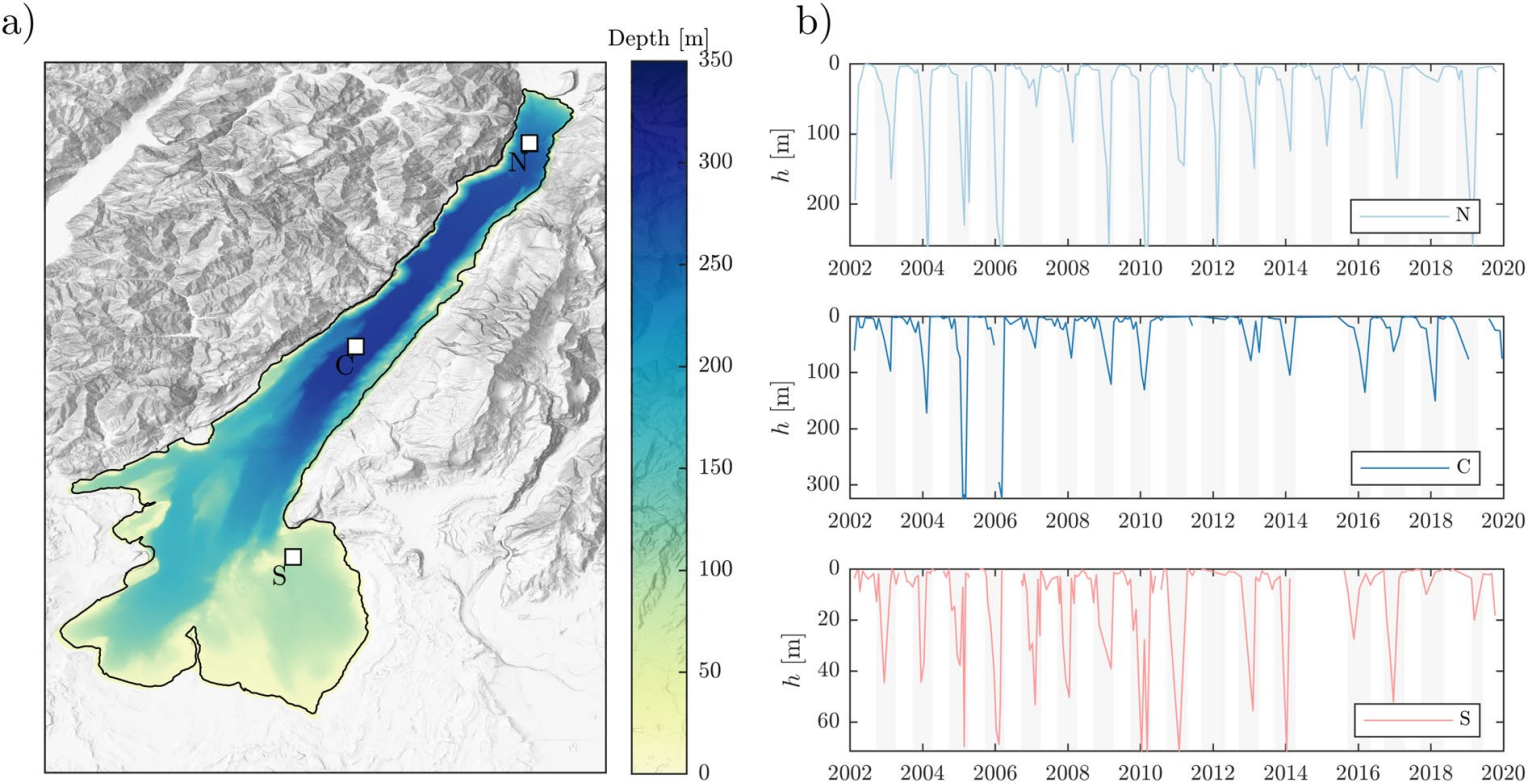
(**a**) Map of Lake Garda with location of in-situ monitoring points (white squares), bathymetry from the latest (2022) high resolution survey from the Hydrographic Institute of the Italian Navy (IIM), and orography of the surrounding region (topographic map credits: ESRItopo^29^). The map shows how the northern part of the lake is flanked by steep slopes. (**b**) Mixed layer depth *h* estimated from in-situ monthly thermal profiles located as in (**a**). Light grey boxes indicate unstratified conditions, i.e, the months when temperature gradients between surface and deeper layers are below 1 ^∘^C. Further information on the climatology of mixed layer depth in the monitoring points is provided in the Supplementary Material).

Our test site is the perialpine and largest Italian Lake Garda (Fig. 1), located in Northern Italy at the altitude of 65 m above the sea level and with a surface extension of 368 km^2^. Its glacial origin determined a narrow northern canyon valley (maximum depth 350 m) nested to a wider and shallower southern basin (maximum depth 80 m).

Lake Garda is classified as an oligomictic lake, despite heterogeneity exists between the two basins. The 0–100 m layer indeed generally undergoes homogeneous cooling every year between December and April (see station S in Fig. 1b) thus leading to a monomictic regime in the southeastern basin. Complete overturn events instead occur occasionally in the deepest part of the lake, with a significant reduction of their frequency in the last decade. The last deep mixing event in Lake Garda dates back to 2006 (see station C in Fig. 1b). From 2007 to present, the lake experienced the longest period of meromixis ever recorded since 1990 according to^30^, who ascribed such a shift in the mixing dynamics to climate change.

Historical in-situ data of water temperature for this lake are available from three monitoring points located in the northern and southern basin (see Fig. 1). Data in the northernmost point (N) are provided by the Environmental Protection Agencies (EPAs) of the Autonomous Province of Trento (APPA) on a monthly basis since 1990. Data in the central (C) and southern (S) stations are collected by the EPA of Veneto Region (ARPAV) on a monthly basis since 2002 with a gap in 2015. Data consist in vertical profiles of temperature available at a monthly/bi-monthly basis. For consistency among in-situ timeseries and with the satellite products timeseries, we selected data from 2002 to 2020 in all monitoring points.

The remote sensing products for Lake Garda were obtained from the European Space Agency (ESA)-Climate Change Initiative (ESA-CCI) Essential Climate Variable (ECV) Lakes, version 2.0.2. The dataset provides several products obtained from multi-sensors satellite observations over the period 1992–2020 and is financed by the European Space Agency (ESA) under the Climate Change Initiative. Among the entire dataset for Lake Garda, we used lake surface water temperature (LSWT), chlorophyll-a (chl-a) and turbidity products. The products are provided with an estimate of uncertainty and guidelines for their use for research activities^31^. We defined our selection/discard criteria in order to find the best compromise between the data availability and the gap-filling needed for a robust statistical analysis. Images having more than a defined percentage of “good quality” pixels were selected, with the threshold for “good quality” and the acceptable percentage varying among the three variables. We refer to Supplementary Material for more detail on the data availability for the case study and on selection criteria adopted. A nearest neighbour interpolation was performed on each image to fill the gaps due to clouds or discarded bad quality pixels, such that full maps of LSWT, chl-a and turbidity were reconstructed. For each quantity, a time series of maps was obtained with uneven time spacing due to the different availability of products from different sensors. We finally obtained an evenly spaced time series with 2(10) days temporal resolution for LSWT (chl-a and turbidity) by taking an average every 2(10) days and linearly interpolating for gapfilling. We pre-processed the data such that each pixel of the maps has zero mean and no seasonal variability. If this is not done, any statistical analysis would result in dominant linear trends (e.g. climate-induced warming from LSWT^32^) and seasonal modulation, thus masking the processes associated with a smaller, yet interesting, variance. Anomalies are obtained by (i) de-trending and (ii) de-seasoning (i.e. subtracting the monthly average from the whole time series) these quantities in each pixel of the maps. The pre-processing steps from the raw signal to the final stationary signal are displayed for each variable in the form of time series for each pixel, climatology and power spectra in the Supplementary Material.

### Estimation of mixed layer depth

To apply the theory described in the preceding sections, we consider only the periods when a relatively constant stratification exists. Based on the climatology of mixed layer depth from in-situ data (available in the Supplementary Material), months from May to September show (on average) a fairly constant *h*, with a discrete time derivative smaller than 0.2 m/day for about 70$\%$ of the time-series in all stations. Thus, we considered daily LSWT anomalies from spring to summer months from 2007 to 2020. As we have discrete data at time points $t_n$, with sampling time $\Delta t$, we have to use the time-discrete version of the Ornstein–Uhlenbeck process which is the first order autoregressive model (AR1 process). We use the notation $X^{n}$ for the LSWT anomaly $\tilde{T}_w$ at time $t_n$, so the AR1 process is written as

8 $$\begin{aligned} X^{n+1} = \phi X^{n} + Z^{n+1}; ~ \phi = e^{-\gamma \Delta t} \end{aligned}$$

Here, $Z^{n+1}$ is a Gaussian random variable with zero mean, variance $\sigma ^2$, while $\phi \in (0,1)$ is the auto-correlation coefficient. The auto-correlation function $r_k$ between observations $k \Delta t$ apart is given by

9 $$\begin{aligned} r_k = \phi ^{k} = e^{k \ln \phi }, \end{aligned}$$

and hence $\phi$ can be directly determined from the $\tilde{T}_w$ time series once it is verified that the autocorrelation indeed displays the behaviour as in Eq. (9).

Once $\phi$ is determined (and hence $\gamma$), the value of $\sigma$ can be determined by computing the spectra of the LSWT anomalies, following Eq. (7). The spectral analysis is also useful to the additional verification that LSWT anomalies have a red noise behaviour. In order to verify that this condition applies also to our signal, we compare the spectrum of LSWT anomalies with that of a synthetic AR1 process built as in Eq. (8) and based on the coefficient $\gamma$ estimated from the autocorrelation fitting. Given the dependence of any AR1 process on the random variable *Z*, the fitted AR1 is built by averaging among 100 possible realisations of the same process and considering the 95$\%$ confidence interval.

Once $\gamma$ is obtained from the autocorrelation and spectral analysis of LSWT anomalies, the mixed layer depth can be calculated from $\gamma$ and the coefficient $\alpha$. We estimate the spatially varying $\alpha$ from modelling results of a calibrated long-term simulation of Lake Garda^33^.

The mixed layer depth is also calculated from in-situ water temperature profiles (see Fig. 1b) for comparison with the scale derived from LSWT anomalies. Being aware of several uncertainties associated with a single definition of the mixed layer depth^3,4^, we calculate *h* as the depth where the thermal difference between surface and deeper water is less than 0.1 ^∘^C. We limit our definition of *h* to those periods when we consider the water column as stratified, i.e. when the difference between the maximum and minimum water temperature is larger than 1 ^∘^C. This makes sense in the oligomictic and deep Lake Garda where a totally unstratified profile rarely develops, despite the temperature gradients between surface and deeper layers are below 1 ^∘^C every late winter-early spring^34,35^.

### EOF analysis

To investigate spatial patterns of variability in Lake Garda, we perform an Empirical Orthogonal Functions (EOF) analysis on water quality variables (LSWT, chl-a and turbidity). The analysis consists of a decomposition of a given variable *Y*(*x*, *y*, *t*) depending on time *t* and space *x*, *y* as

10 $$\begin{aligned} Y(x,y,t) = \sum _{l = 1}^{L}{PC_l(t) EOF_l(x,y)} \end{aligned}$$

Such a decomposition allows determining a set of *L* orthogonal functions which have spatial structures and an amplitude that varies in time. Hence, for each mode it is possible to evaluate patterns in the domain of space (typically referred to as EOF) and time (typically referred to as expansion coefficients or EOF amplitude or principal components PC). The combination of the obtained EOFs and PCs from this decomposition allows reconstructing the original time series as in Eq. (10).

For the computation of dominant EOFs in the anomalies of LSWT, chl-a and turbidity, we also remove the spatial mean from each map of the time series. This preliminary step is necessary due to the limited size of the lake’s domain: if the spatial mean is maintained, the first EOF presents a dominant monopole pattern whose corresponding PC1 resembles the time variation of the spatial mean. Thus, removing the spatial mean allows discarding from our analysis those dynamics that trigger a homogeneous response of the lake (e.g. larger scale interannual variability) and focusing on what drives the internal variability of the lake’s features.

We report the EOF analysis results in terms of correlation maps between the original variables (detrended and deseasoned time series, henceforth anomalies). We prefer this visualization, rather than the traditional eigenvalue maps, as it eases the interpretation of the results in terms of spatial correlation and anti-correlation among the different areas of the lake. Areas showing the same correlation sign can be interpreted as presenting the same behaviour with respect to the process described by the EOFs. Together with the correlation maps, we also report maps describing the areas of the lake where each mode is mostly responsible of the quantity’s variance.

## Results

### Mixed layer depth from LSWT anomaly

**Figure 2:**
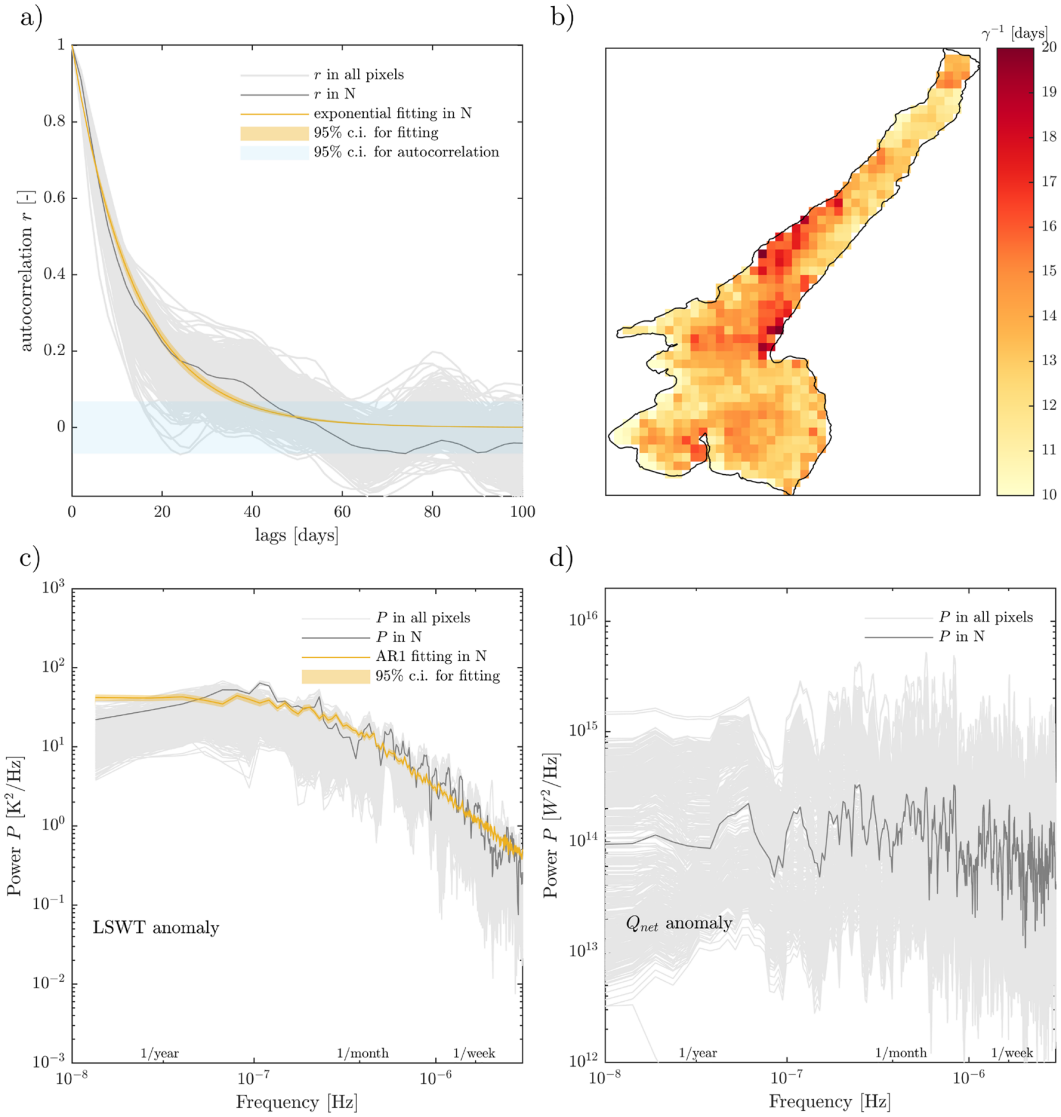
(**a**) Auto-correlation of LSWT anomaly at N (thick grey line, see Fig. 1 for the location) and in all other pixels (thin grey lines). Exponential fitting of *r* at N (orange line) and corresponding 95$\%$ confidence bounds (orange shadowed area). The horizontal light blue box shows the 95$\%$ confidence bounds considered for the computation of auto-correlation. (**b**) Spatial variability of the damping time scale (inverse of $\gamma$) obtained from exponential fitting in each pixel of the map. (**c**) Power spectra of LSWT anomalies in N (thick grey line) and in all other pixels (thin grey lines). Spectrum of a synthetic AR1 process computed at N (orange line) by considering $\gamma =0.0725 d^{-1}$ ($\sim$ 14 days) and 95$\%$ confidence bounds (orange shadowed area) from 100 realisations. (**d**) White spectrum of net heat flux anomalies based on Delft3D model simulations on Lake Garda in all model pixels (thin grey lines) and in N (thick grey line).

In Fig. 2a, the auto-correlation function *r*, computed pixel by pixel, is shown together with an example of exponential fitting based on Eq. (9) for one pixel, corresponding to the measuring point N in Fig. 1.

The auto-correlation of LSWT anomalies rapidly decreases with *k* (in days) from $r_0 = 1$. In most pixels, $r = e^{-1}$ between 10 and 30 days, while going below the confidence level 95$\%$ or directly to 0 between 30 and 70 days. Such decay is clearly exponential and can be approximated with a function of the kind in Eq. (9). The obtained time scale $\gamma ^{-1}$ has a clear spatial pattern (Fig. 2b) suggesting that heterogeneity exists in the lake in terms of thermal inertia. The noisiest areas are found in the northern (10 ± 1 days) and southern (10 ± 2 days) area, with a gradient of persistence time from the periphery to the centre of the lake, where maximum values reach 20 ± 1 days. The uncertainty associated with the exponential fitting (ranging from 1 to 2 days) is thus much smaller than the spatial variability observed (10–20 days) and the fitting performs well in most pixels, with $R^2 > 0.9$ everywhere in the lake. Maps of $R^2$ and uncertainty on $\gamma ^{-1}$ estimate are provided in Supplementary Material.

The spectrum of the LSWT anomalies (Fig. 2c) appears flat around a more or less constant power level (5 $\times 10^{1} {\text {K}}^2/{\text {Hz}}$) at frequencies associated with temporal scales longer than three months ($10^{-7} {\text {Hz}}$). The power associated with higher frequencies (1 month to a few days) decreases rapidly by 2 orders of magnitude (from $10^{1}$ to $10^{-1}$ ${\text {K}}^2/{\text {Hz}}$) in the example pixel. The AR1 process simulated based on estimated $\gamma$ in that pixel (see Fig. 2a) overlaps with the sample spectrum, confirming that its behaviour can be well approximated by an auto-regressive process. The power spectrum of the net heat flux (Fig. 2d) shows instead the same order of magnitude ($10^{-14} {\text {W}}^2/{\text {Hz}}$) in the full range of frequencies from 1 year to 1 day, resembling white noise behaviour.

**Figure 3:**
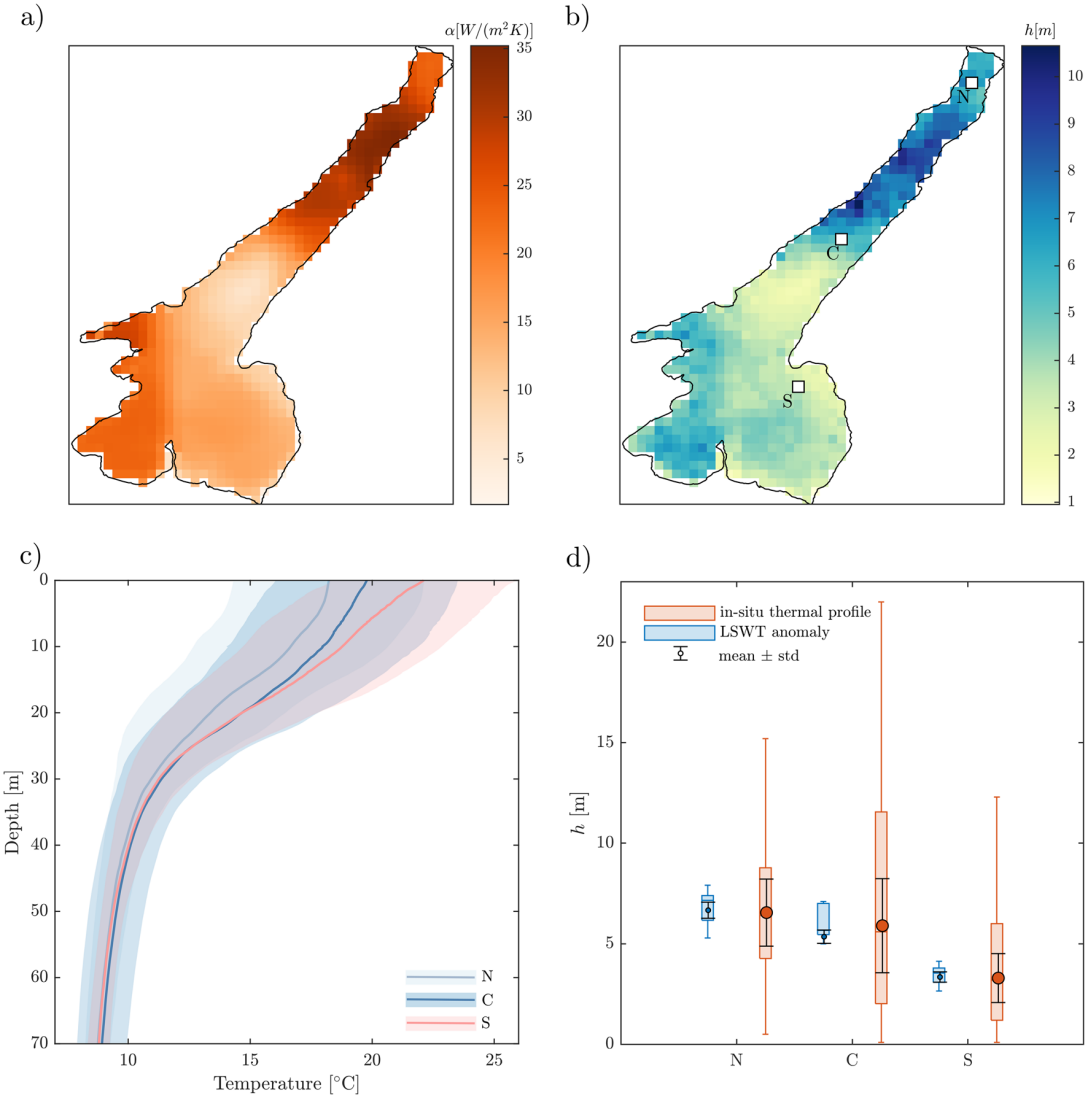
(**a**) Spatial variability of the coefficient $\alpha$ based on linear regression of $Q_{net}$ anomaly as a function of $T_a - T_w$ anomaly in each grid cell of Delft3D model simulations on Lake Garda^33^; (**b**) spatial variability of *h* estimated by inverting Eq. (3). The white squares identify the pixels closest to in-situ monitoring points and considered for panel (**d**); (**c**) Water temperature profiles at the three monitoring stations during the stratified months (from May to September). Shaded area shows the standard deviation and the line the average over the period 2002–2020; (**d**) Estimation of mixed layer depth *h* from LSWT anomaly (blue boxes) and in-situ measurements (orange boxes) at the three monitoring points. Blue boxes represent the spatial variability within a 3 by 3 pixels area of *h* as estimated by inverting Eq. (3) with $\gamma$ from Fig. 2. Orange boxes represent the temporal variability of *h* as computed from the timeseries of temperature profiles (2002–2020) assuming a threshold temperature between surface and interior layer of 0.1^∘^. The median, the lower and upper quartiles of both estimates are shown as the orange/blue line inside the box, the bottom and top edges of the box respectively. The whiskers endpoints show the shallowest and deepest *h* from the sample. Errorbars overlapped on boxes show the uncertainty associated with the computation method: 95% confidence interval from the fitting of $\gamma$ for blue boxes; use of different threshold temperature gradient (from 0.1 to 1 ^∘^C) for orange boxes.

Together with the damping time scale, the linear coefficient $\alpha$ contributes determining spatial variability of the surface mixed layer response to atmospheric forcing. As shown in Fig. 3a, a stronger dependence of net heat flux anomalies on air-water thermal gradients characterise the upper part of the lake based on the three-dimensional model^33^. The value of $\alpha$ ranges between 10 to 35 ${\text {W}} {\text {m}}^{-2} {\text {K}}^{-2}$ across the lake, with a mean value of 18 ${\text {W}} {\text {m}}^{-2} {\text {K}}^{-2}$. Such spatial variability, combined with that of the damping time scale $\gamma ^{-1}$, leads to the mixed layer depth estimate shown in Fig. 3b. Deeper *h* are found in the northern-central basin, with $h = 7.18 \pm 0.3$ m at station N and $h = 5.29 \pm 0.27$ m at C. In the southern part, $h = 3.18 \pm 0.24$ m is the value close to station S. The estimate of *h* slightly varies within a radius of 3 km from the pixel closest to in-situ monitoring stations (blue boxes in Fig. 3d), but the variability has the same order of magnitude of the uncertainty associated with the fitting of $\gamma$ (errorbars). Such estimates are consistent with the range of variability of *h* computed from in-situ data (Fig. 3c,d) in the stratified months. From N to S the average shape of the profile (Fig. 3c) is gradually modified in the surface 30 m from a well-defined mixed layer (in N) to the almost linear temperature decrease along depth at S, where a mixed layer can hardly be identified.

In all stations, the estimate of *h* from LSWT anomaly is consistent with the median values obtained from in-situ thermal profiles (Fig. 3d) and confirms that the surface mixed layer is generally shallower and less variable in the southern basin (S) compared to the northern one (N,C). We finally note that the mean value of *h* estimated from in-situ data also depends on the threshold used for determining the mixed layer depth (black errorbars overlapped to in-situ estimates in Fig. 3d).

### Dominant modes of spatio-temporal variability in water quality quantities

**Figure 4:**
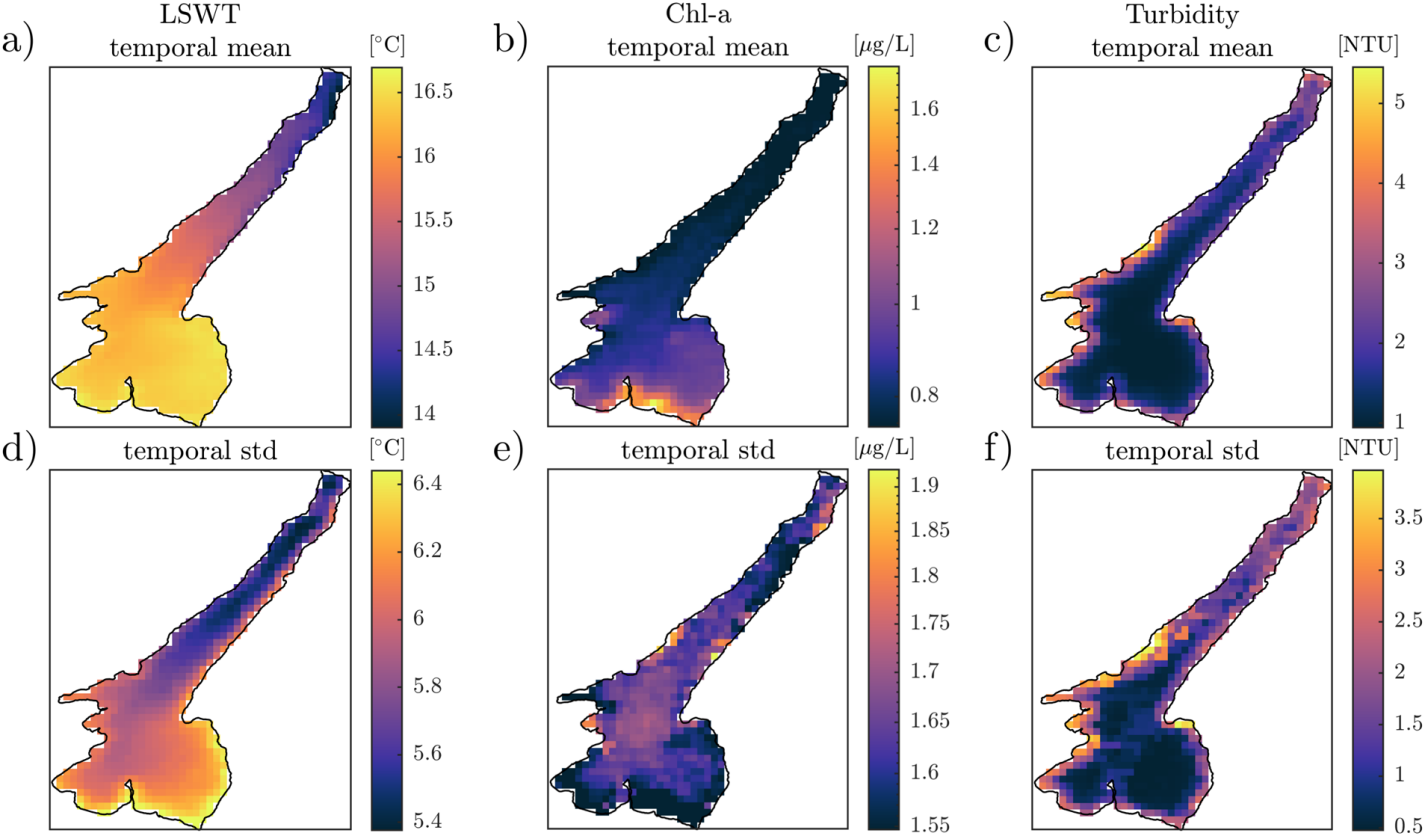
Spatial distribution of temporal mean (top, **a**–**c**) and standard deviation (bottom, **d**–**f**) of LSWT (**a**,**d**), chl-a (**b**,**e**) and turbidity (**c**,**f**) in Lake Garda. Mean and standard deviations are computed on the gap-filled time series from 2007 to 2020 for LSWT (2 days timestep), and from 2003 to 2012 for chl-a and turbidity (10 days timestep).

The mean and standard deviation of LSWT, chl-a and turbidity timeseries for the case study is spatially displayed in Fig. 4.

The map of temporal mean of LSWT (a) shows a dominant north-south gradient with largest temperatures associated, on average, to the southern basin and coldest temperatures to the northern basin. The standard deviation map (d) reveals instead a marked east-west gradient, with the eastern and shallow part of the lake tending to be subject to higher variability. Chl-a concentration (b) tends to be very low in the northern basin and higher in the southern, with values along the southern shore being on average more than twice those in the pelagic areas of the lake, where variability is higher instead (e). Finally, turbidity (c) shows a strong difference between coastal and pelagic zones on average, with shores tending to be way more turbid (and more subject to variability) than open waters.

**Figure 5:**
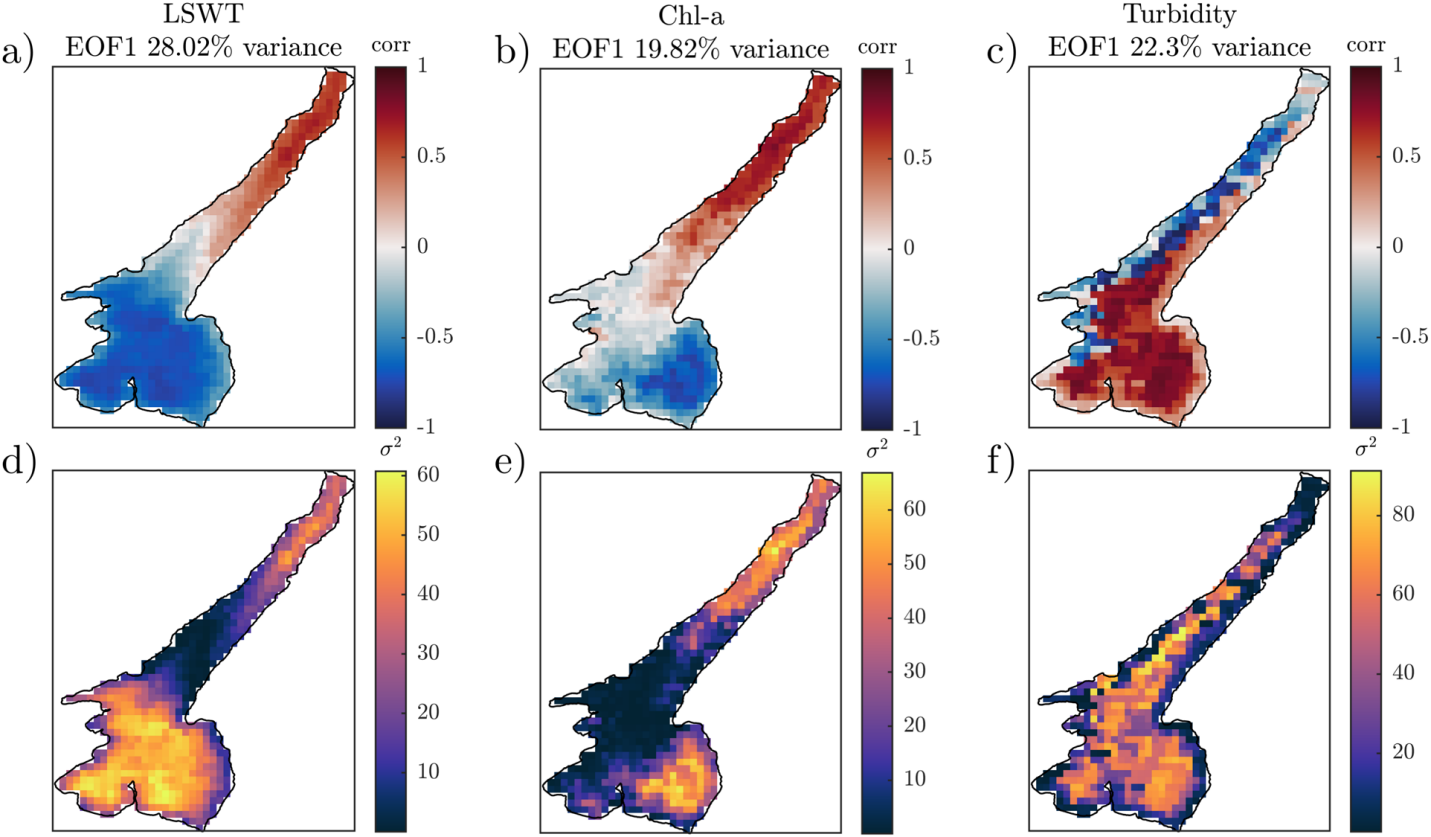
Correlation (top) and variance (bottom) associated with the first dominant EOF of LSWT (**a**,**d**), chl-a (**b**,**e**) and turbidity (**c**,**f**) anomalies in Lake Garda.

In Fig. 5 we present the dominant patterns of variability for these quantities as resulted from the EOF analysis. The first three dominant modes (EOF1, EOF2 and EOF3) are included in the Supplementary Material. We focus here on EOF1 and the explained variance associated to it for each of the analysed time series. The first EOF of LSWT (a) and chl-a (b) maps shows a dipole pattern discriminating the response of the northern and southern basins. White pixels define the front of this pattern and lie along the physical boundary (see Fig. 1) between the two basins. The transition between the two anti-correlated areas indeed occurs at the end of the longitudinal axis of the lake, where the deep and elongated canyon gives way to the shallow and round-shaped southern basin. The first EOF mode explains 28.02$\%$ of the total variance of LSWT and 19.82$\%$ of chl-a anomalies. Moreover, EOF1 appears to be responsible of up to 70$\%$ of variance of LSWT and chl-a anomaly in the southeastern basin and a smaller but significant percentage (about 40$\%$) of variance of the northwestern basin. EOF1 of turbidity anomaly (c) explains 22.3$\%$ of variance and shows a different pattern from that of LSWT and chl-a. The interface between the two anti-correlated basins in turbidity EOF1 is shifted towards west and identifies a clear east-west gradient. Moreover, panel f clarifies that this EOF1 explains the variance of turbidity anomaly in the pelagic areas of the lake without preferential areas. The observed patterns of LSWT EOF1 and chl-a EOF1 are statistically correlated one other with a cross correlation coefficient of 0.83 and with mixed layer depth pattern retrieved from LSWT anomaly (see Fig. 3b) with 0.74 (LSWT EOF1-*h*) and 0.77 (chl-a EOF1-*h*). Weaker correlation is found between the spatial patterns of turbidity EOF1 and LSWT EOF1, chl-a EOF1 and *h* (0.31, 0.41 and 0.36, respectively).

## Discussion and conclusions

Results from the application of the stochastic climate model by^22^ have shown that spatial gradients of the temporal time scale $\gamma ^{-1}$ and *h* exist at basin-scale in Lake Garda. Such different thermal inertia is also reflected by the dominant mode of LSWT and chl-a variability obtained via the EOF analysis. Mixed layer depth variability indeed affects both surface temperature and phytoplankton dynamics, while not having necessarily significant impacts on turbidity in a clear water environment as Lake Garda. In this lake chl-a concentration is not strongly correlated to turbidity (as clearly visible in Fig. 4), unless for extreme bloom events, because its waters are “optically complex” and fall into a Case 2 definition by^36,37^. While Case 1 generally applies to open ocean waters, whose optical properties are dominated by phytoplankton, waters classified as Case 2 generally have optical properties influenced by other constituents, such as CDOM and inorganic mineral particles. The most important characteristic of Class 2 waters is that the constituents determining their optical properties do not covary with the phytoplankton concentration^38^. As many years of further investigations clarified, these are ideal cases not necessarily encountered in nature, and many intermediate cases can exist even in the same study site, due to temporal (e.g. seasonal) or spatial variability of the processes causing the optical water properties. However, most lacustrine environments fall into Case 2 definition, including the clear blue Lake Garda. This lake generally shows high values of water transparency (10 m average Secchi depth^30^), mean chl-a concentrations of 2–4 mg m^-3^^39^ with minima of 0.5 and very rare maxima of about 12 mg m^-3^ during occasional fall cyanobacteria blooms^40^. Concentrations of suspended sediments instead are less variable and range from 0.1 to 5.5 g m^-3^, with maxima along the shore due to erosion and run-off from the catchment, generally after precipitation events^41^, or in pelagic areas after turbidity discharges^42^, or wind-induced resuspension of the bottom sediments.

Morphological differences in Lake Garda have already been associated with different thermal regimes and trophic conditions from in-situ observations^43,44^, remote sensing^45^ and modelling^33,34^ studies. In good agreement with past studies addressing Lake Garda mixing regime in the context of climate change^30,39,43^, we demonstrate that the spatial heterogeneity found in this lake, which is common to many multi-basin lakes (e.g. Lake Lugano^46^), depends on basin-scale variability of both thermal inertia (expressed by $\gamma ^{-1}$), and net heat fluxes with the atmosphere (expressed by $\alpha$). The latter integrates spatially varying atmospheric variables, in particular wind, air temperature and relative humidity, whose spatial patterns were already found to affect basin-scale variability of hourly and daily evaporation rates in this lake^47^.

The relation between LSWT anomalies persistence time and local depth was observed by^25,26^ in many lakes at the Northern Hemisphere during the stratified season. Similarly to our findings, the authors studied the temporal persistence of LSWT anomalies and ascribed longer persistence time to deeper lakes or deeper regions of single lakes. Our result complement their conclusions on the timing and duration of thermal stratification by proving that such persistence time is carrying the information on the vertical extent of surface mixed layer at basin-scale and by quantifying such information from a simple statistical approach. Our findings are also in good agreement with those from^10^, who provided a formulation for the thermally reactive layer as a function of bulk LSWT. Their approach indeed implicitly relies on the statistical properties of LSWT anomalies we demonstrated in this work for the first time in a lake. On this regard we note that linking the extent of surface mixed layer to the de-correlation time scales of LSWT anomalies ($\gamma ^{-1}$) provides a valuable a-priori information also for the optimal application of data-driven models as those explored by^48^ for the prediction of LSWT. The order of magnitude of *h* we obtained via stochastic modelling is comparable to that from in-situ thermal profiles if a threshold thermal gradient of 0.1 ^∘^C is set between the surface and the deeper layers. Such threshold is consistent with the accuracy standards from the EPAs in charge of water quality monitoring in our case study^49^ and with other studies addressing similar topics^10^. The layer of water that shows a red noise response to atmospheric variability is a layer actively responding to short term atmospheric variability and carrying the memory of past mixing events within a time scale $\gamma ^{-1}$. The order of magnitude of the obtained $\gamma ^{-1}$ for our case study shows that we are looking at a layer whose temperature anomaly may persist for a period of 1–3 weeks. Such layer is characterised by weak thermal stratification and homogeneous turbulence^5^.

It is clear that the order of magnitude of both $\gamma ^{-1}$ and *h* can be strongly affected by the data availability of the original LSWT time series. As the estimation of *h* is directly related to $\gamma ^{-1}$, large uncertainty on this value is expected for timeseries whose sampling frequency approaches or exceeds the physically meaningful $\gamma ^{-1}$. For the case study, we verified that, on a spatial average, consistent results can be obtained if the timeseries of LSWT is resampled up to 10 days. For sampling frequencies equal to 15 days (corresponding to the spatial average of $\gamma ^{-1}$ in Fig. 2b), the uncertainty is maximal, while for larger values the estimated timescale is equal to the sampling frequency itself, as autocorrelation is lost after only one timestep. We refer the reader to the Supplementary Material for more insights on uncertainty and mixed layer depth estimates for different sampling intervals. This poses a physical limit to the application of this theory in any context where data is not available at daily or a few days time interval but also provides useful insights into the physical consistency of gap-filling procedures for such contexts. The de-correlation time scale indeed defines a limit to the persistence, hence the predictability, of the observed anomaly^50^, physically constraining also gap-filling procedures. The knowledge of the spatial variability of autocorrelation decay time of LSWT anomalies might provide the necessary theoretical background for a physically- consistent, but yet simpler, autoregressive gap-filling.

We have shown that the spatial distribution of *h* resembles that of the first dominant mode of LSWT and chl-a. Many factors contribute to the statistical correlation between LSWT and chl-a. Aside from direct effects of water temperature on phytoplankton abundance (related to e.g. temperature-dependent metabolism and nutrient-cycling), horizontal and vertical advection can drive the surface patterns of both LSWT and chl-a. On this issue,^51^ showed that positive and negative correlation between the spatio-temporal patterns of LSWT and chl-a may also depend on the season and on localised short-term up/downwelling altering water temperature and nutrients availability within the euphotic zone. However, phytoplankton abundance also depends on turbulence and stratification in the surface mixed layer^52^. Strong stratification (i.e. shallower *h*) indeed confines phytoplankton to the surface shallow layer, where light is abundant, and promote growth, while a weaker stratification (i.e. deeper *h*) allows deeper mixing, thus diluting phytoplankton communities^53^. It is not therefore surprising that, in our case study, areas showing the highest mean chl-a concentrations (southeastern basin, Fig. 4) also show the shallowest *h* (Fig. 3b), while the northern lake is typically less productive, subject to higher disturbance from the atmosphere (Fig. 3a), and less stratified (deeper *h*). The results from this single case study suggest that how fast chl-a responds to short-term climate alterations might depend on local depth as well as water temperature. In agreement with^53^, and well in line with our findings,^54^ have shown that the timing of chl-a peaks to heatwaves was faster in shallow lakes than in medium and deep lakes. On this regard, the response of chl-a to atmospheric disturbance might be investigated with an approach similar to the one proposed for interpreting LSWT anomalies. Considering that chl-a anomalies show a red-noise spectrum in Lake Garda as well (see Supplementary Material), a double integration approach could be applied to interpret the chl-a anomalies as proposed by^55^ for zooplankton communities.

Finally, defining lakes as integrators of atmospheric variability is a first-order approximation of far more complex interactions between physical and biological processes occurring in the surface mixed layer. Given the simplicity of the proposed approach, its extension to the global scale is quite straightforward. A few limitations apply to such an extension. The theory behind this study can be applied to lakes that show a more or less prolonged period of thermal stratification, for which the definition of mixed layer depth holds. This requirement automatically excludes lakes mixed to the bottom for most of the year (i.e. polymictic lakes). On this aspect we recommend the physically sound scaling criterion by^2^, who proposed a critical mean basin depth to discriminate between the two main mixing regimes. More obvious limitations are related to the data availability. On this regard, global datasets complementary to ESA-CCI should be explored to provide the necessary information (e.g. reanalysis data for air-water heat fluxes), given that the temporal and spatial scales of the lake are adequately described by both temporal and spatial resolution of the adopted datasets. At the scale of our case study, such approximation allowed associating the principal stochastic properties of LSWT anomalies to the basin-scale variability of the surface mixed layer. This first-order picture necessarily overlooks finer scale dynamics, e.g. the effect of intense currents advecting water temperature vertically and horizontally, seasonal modulations and higher order modes of variability, which can be well described by more complex deterministic models. However, the lessons learnt from weather and climate modelling is that a significant gain in prediction capabilities can be achieved by combining a stochastic approach to deterministic models^56^. In this context, we believe that our findings open a new perspective for climate change-oriented investigations in the field of inland waters.

### Supplementary Information

Supplementary Information.

## Data Availability

The ESA Climate Change Initiative (CCI) lakes dataset analysed during the current study is available at https://data.ceda.ac.uk/neodc/esacci/lakes/data/lake_products/L3S/v2.0.2. Timeseries for the case study were extracted with the python extraction scripts at https://github.com/elisaca/LakeCREST. In-situ water temperature profiles are provided by the Environmental Protection Agency of Veneto Region (ARPAV) and Autonomous Province of Trento (APPA). The high resolution bathymetry of Lake Garda was provided by the Hydrographic Institute of the Italian Navy (IIM) and realized within the project ACCURATE (https://asa.unicatt.it/asa-progetti-accurate) in collaboration with the Catholic University of Sacred Heart (UNICAT, Brescia). Both in-situ and bathymetric data are available upon request. All steps for the EOF analysis (from preprocessing to final EOF computation) were carried out following^57^. Figure 1a was created using QGIS software using ArcGIS Online basemap^29^, sourced from https://www.arcgis.com/home/item.html?id=7dc6cea0b1764a1f9af2e679f642f0f5 under license. ArcGIS®and ArcMap^TM^ are the intellectual property of Esri and are used herein under license. Copyright ©Esri. All rights reserved. For more information about Esri®software, please visit www.esri.com.
