# Supplementary material for: Slow response of surface water temperature to fast atmospheric variability reveals mixing heterogeneity in a deep lake
Source: Sci Rep. 2024 Apr 11;14:8459. doi: 10.1038/s41598-024-58547-0 (PMC11009398; doi:10.1038/s41598-024-58547-0)
Supplement: Supplementary file 1 — Supplementary Information. [file 41598_2024_58547_MOESM1_ESM.pdf]

Supplementary information for: Lakes surface mixed layers slowly integrate fast atmospheric variability: Evidence from a deep perialpline lake

Marina Amadori[1,*], Mariano Bresciani[1], Claudia Giardino[1], and Henk A. Dijkstra[2]

[1]Institute for Electromagnetic Sensing of the Environment, National Research Council, Milan, 20133, Italy
[2]Institute for Marine and Atmospheric research Utrecht, Department of Physics, Utrecht University, Utrecht, 3584 CC, The Netherlands.
[*]amadori.m@irea.cnr.it

Introduction

In this supplementary information we report:

- Data availability from ESA CCI ECV Lakes dataset for Lake Garda and selection/discard criteria;
- Pre-processing of time series;
- First three dominant modes of LSWT, chl-a and turbidity from EOF analysis;
- Thermal profiles and Climatology of mixed layer depth and Secchi depth from monitoring points;
- Uncertainty associated to mixed layer depth estimation from LSWT anomaly.

Data availability from ESA CCI ECV Lakes dataset for Lake Garda and selection/discard criteria

The remote sensing products for Lake Garda were obtained from version 2.0.2 of the dataset produced under the European Space Agency (ESA) Climate Change Initiative (ESA-CCI), which includes lakes as Essential Climate Variable (ECV). Such dataset offers multi-sensors satellite observations over the period 1992-2020 for several thematic variables and for 2024 lakes. For Lake Garda, data is available from 1995 onwards. In particular we used Lake Surface Water Temperature (LSWT), chlorophyll-a (chl-a) and turbidity products, with related quality flags. Chl-a and turbidity are not ECVs but are provided as estimated from the Lake Water Leaving Reflectance (LWLR) ECV. We followed the Product User Guide[1] for selection good quality thresholds for research purposes. For LSWT products we selected images having more than 70 % of pixels with quality flag ≥ 4. The remote sensing products for Lake Garda were obtained from the European Space Agency (ESA)- Climate Change Initiative (ESA-CCI) Essential Climate Variable (ECV) Lakes, version 2.0.2. The dataset provides several products obtained from multi-sensors satellite observations over the period 1992-2020 and is produced by the European Space Agency (ESA) under the Climate Change Initiative. The images meeting this requirement are 1537. 50% of the images are available on a daily basis and 90% with a time distance of less than 5 days. Largest gaps are found in images acquired before 2007 (see Figure 1). For such a reason we analyzed the timeseries starting from 2007. Among the entire dataset for Lake Garda, we used lake surface water temperature (LSWT), chlorophyll-a (chl-a) and turbidity products. The products are provided with an estimate of uncertainty and guidelines for their use for research activities[?]. We defined our selection/discard criteria in order to find the best compromise between the data availability and the gap-filling needed for a robust statistical analysis. Images having more than a defined percentage of “good quality" pixels were selected, with the threshold for “good quality" and the acceptable percentage varying among the three variables. We refer to Supplementary Material for more detail on the data availability for the case study and on selection criteria adopted. Chl-a and turbidity products are available from 2002 to 2020 with a long gap between 2012 and 2016 which occurred between to the end of the ENVISAT mission (MERIS sensor) and the launch of the new Sentinel-3 mission (OLCI - sensor). A nearest neighbour interpolation was performed on each image to fill the gaps due to clouds or discarded bad quality pixels, such that full maps of LSWT, chl-a and turbidity were reconstructed. For each quantity, a time series of maps was obtained with uneven time spacing due to the different availability of products from different sensors. We finally obtained an evenly spaced time series with 2(10) days temporal resolution for LSWT (chl-a and turbidity) by taking an average every 2(10) days and linearly interpolating for gapfilling. For chloropyll-a (chl-a) products, we selected images having more than 70 % of pixels with uncertainty below 60 %. The images meeting this requirement are 548. More than 50% of the images are available at a time resolution ranging between 1 and 4 days. We pre-processed the data such that each pixel of the maps has zero mean and no seasonal variability. If this is not done, any statistical analysis would result in dominant linear trends (e.g. climate-induced warming from LSWT[?]) and seasonal modulation, thus masking the processes associated with a smaller, yet interesting, variance. Anomalies are obtained by i) de-trending and ii) de-seasoning (i.e. subtracting the monthly average from the whole time series) these quantities in each pixel of the maps. For turbidity products (NTU) images with more than 70 % of pixels with uncertainty below 70 % were selected. The images meeting this requirement are 773. More than 50% of the images are available at a time resolution ranging between 1 and 4 days. For both chl-a and NTU products we analyzed the timeseries between 2003-2012.

[1] Product User Guide CCI-LAKES-0029-PUG_V1.1 https://climate.esa.int/media/documents/CCI-LAKES-0029-PUG_v1.1_signed_CA.pdf

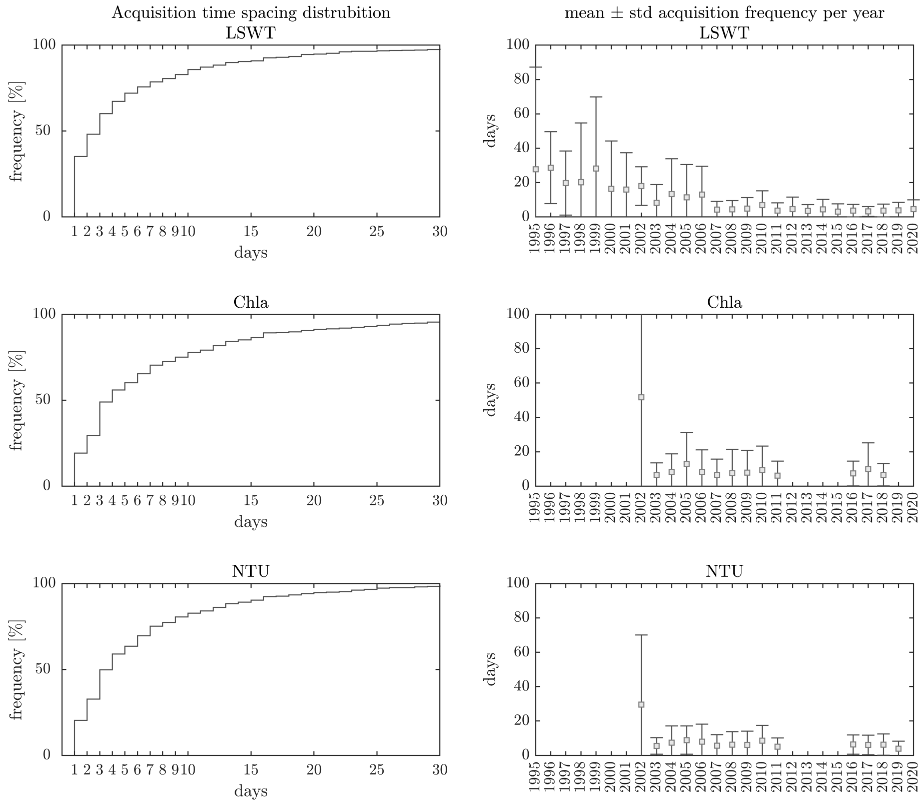

Figure 1. Frequency distribution (left) and yearly statistics (right) of time spacing of the final time series of LSWT, chl-a and turbidity selected from the CCI-Lakes database according to the quality and availability thresholds.

Pre-processing of time series

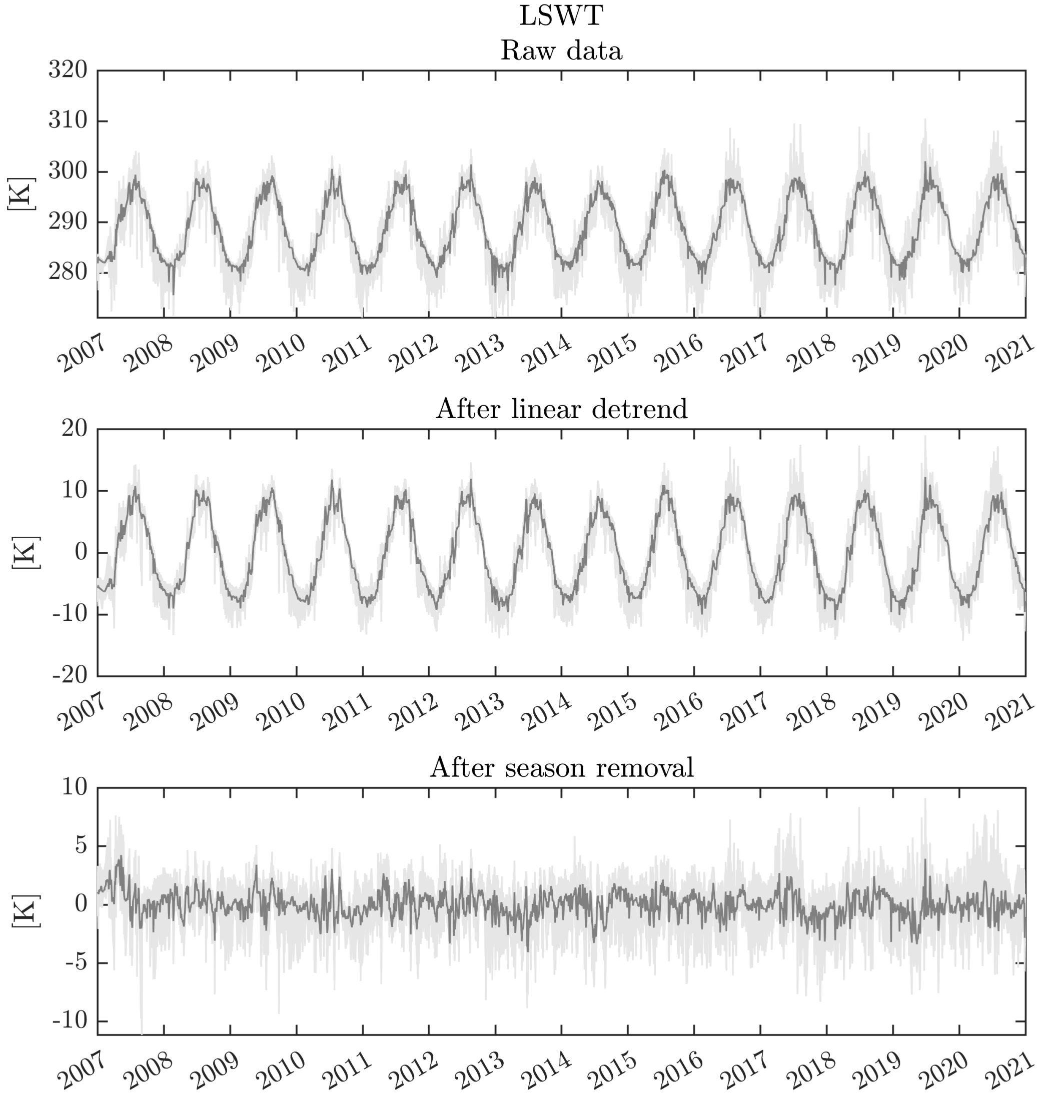

Figure 2. Pre-processing of LSWT timeseries from raw to detrended and deseasoned data. (all pixels: light grey lines, spatial mean: bold dark line)

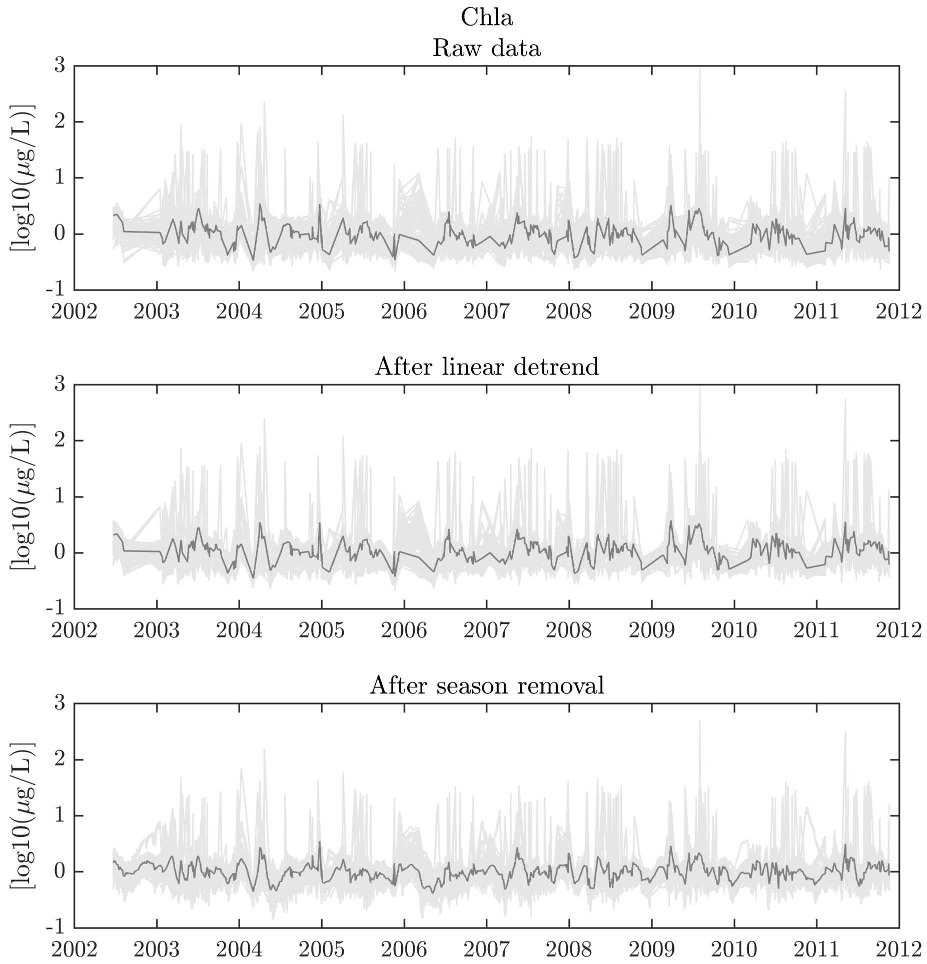

Figure 3. Pre-processing of chl-a timeseries from raw to detrended and deseasoned data. (all pixels: light grey lines, spatial mean: bold dark line)

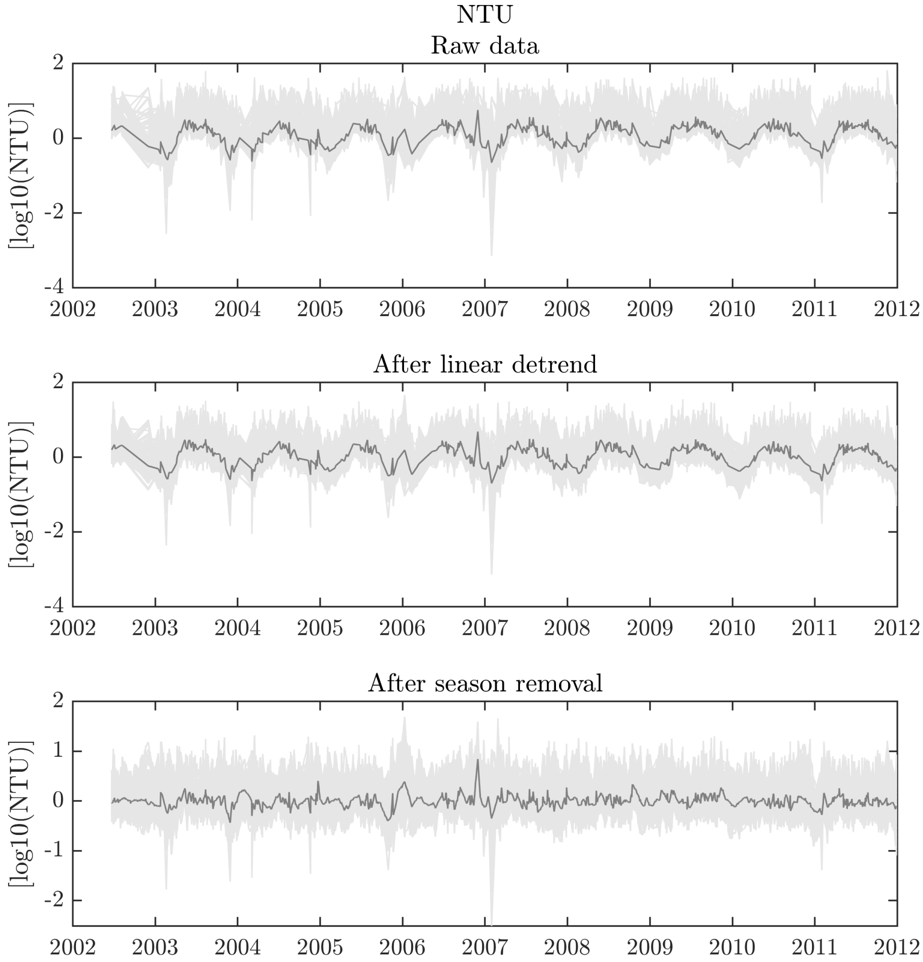

Figure 4. Pre-processing of turbidity timeseries from raw to detrended and deseasoned data. (all pixels: light grey lines, spatial mean: bold dark line)

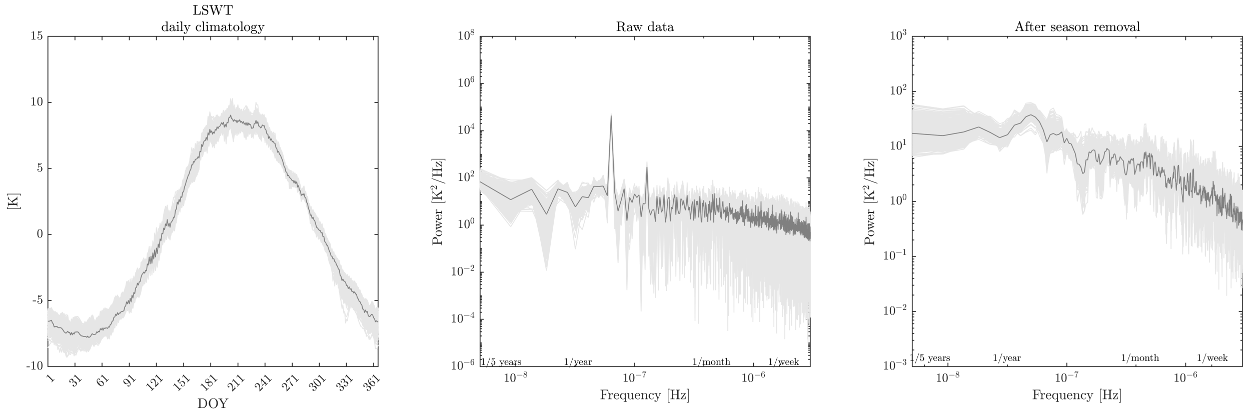

Figure 5. Climatology of LSWT removed for deseasoning procedure (see Fig. 2); power spectrum of raw signal and power spectrum of final pre-processed signal (all pixels: light grey lines, spatial mean: bold dark line). Note: all-year signal is displayed here.

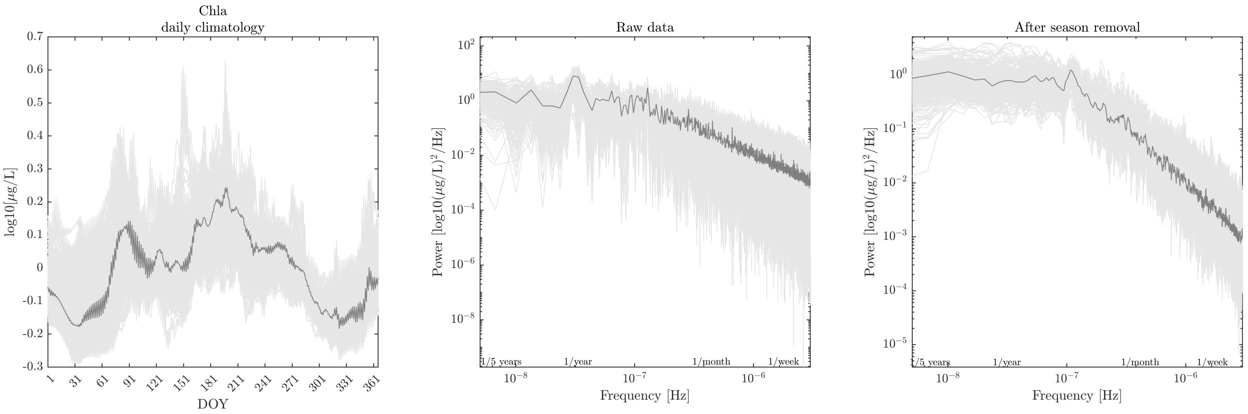

Figure 6. Climatology of chl-a removed for deseasoning procedure (see Fig. 3); power spectrum of raw signal and power spectrum of final pre-processed signal (all pixels: light grey lines, spatial mean: bold dark line).

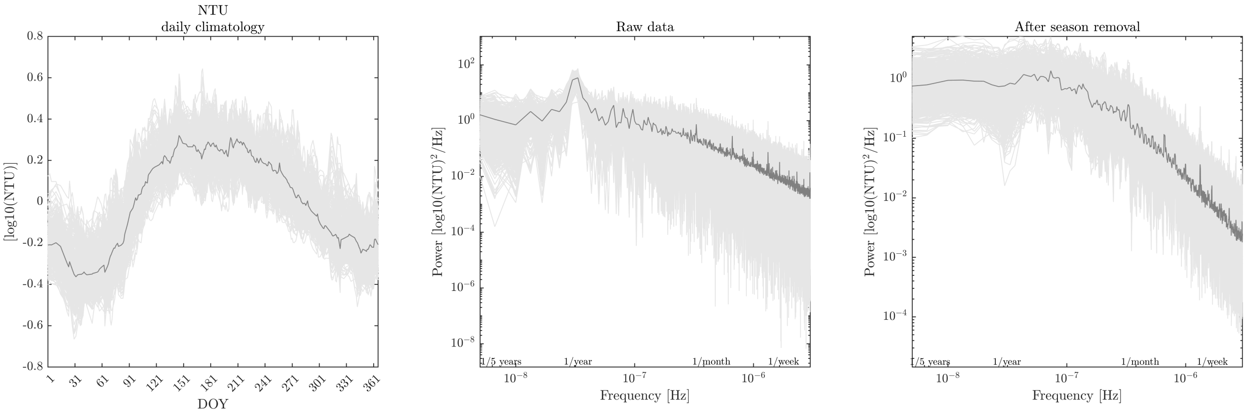

Figure 7. Climatology of turbidity removed for deseasoning procedure (see Fig. 4); power spectrum of raw signal and power spectrum of final pre-processed signal (all pixels: light grey lines, spatial mean: bold dark line).

First three dominant modes of LSWT, chl-a and turbidity from EOF analysis

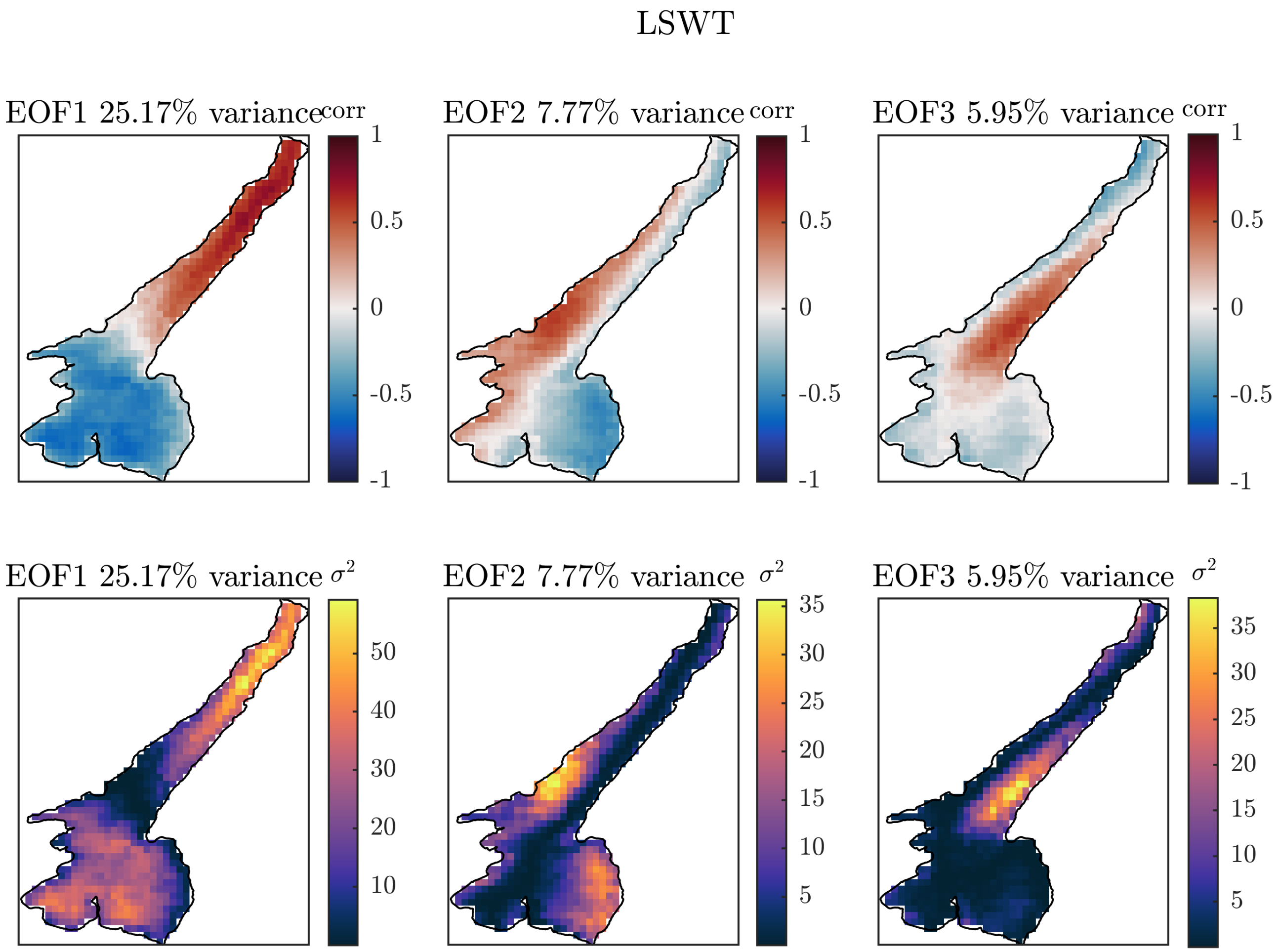

Figure 8. Correlation (top) and variance (bottom) associated to the three dominant EOFs of LSWT in Lake Garda.

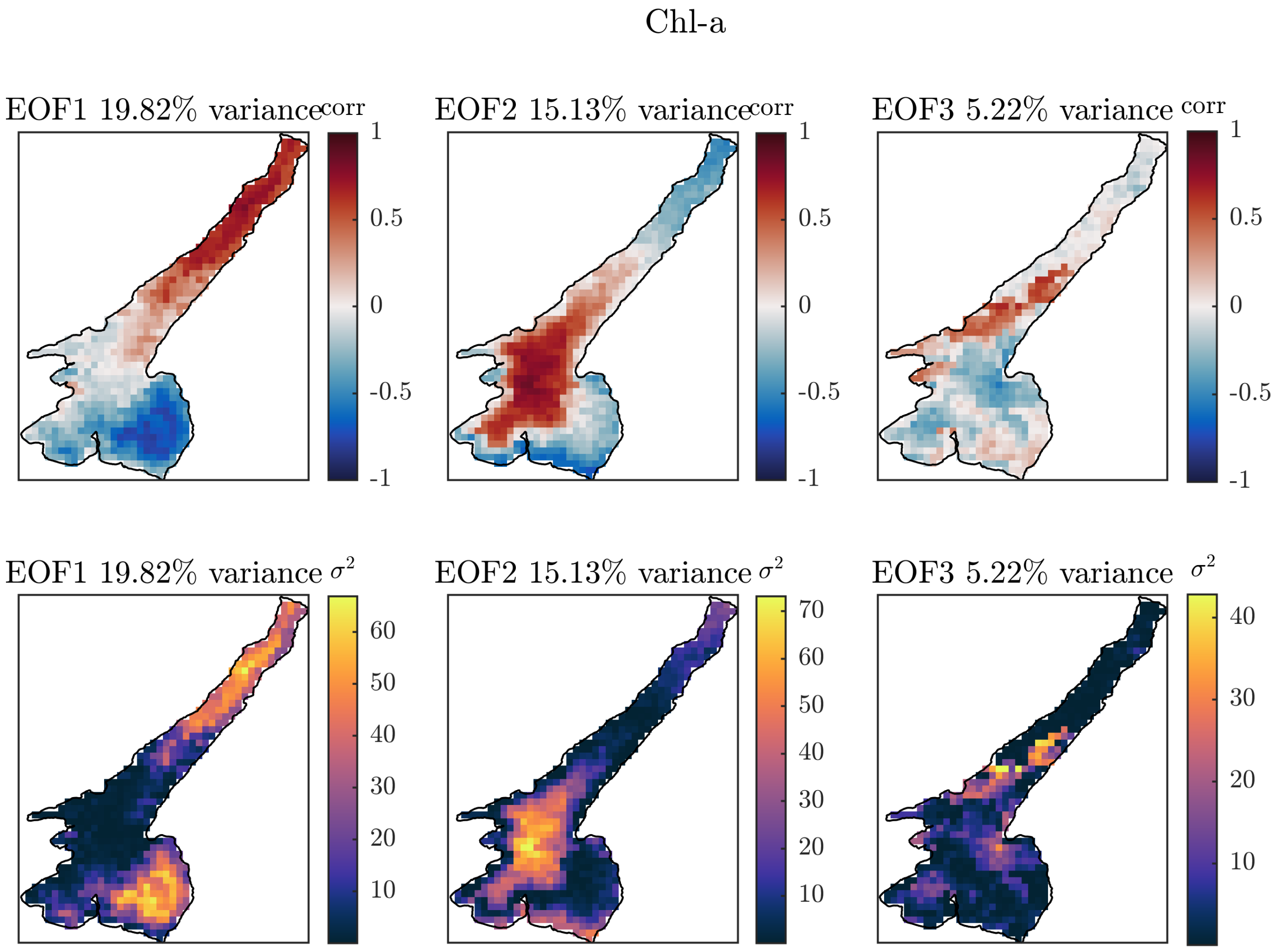

Figure 9. Correlation (top) and variance (bottom) associated to the three dominant EOFs of chl-a in Lake Garda.

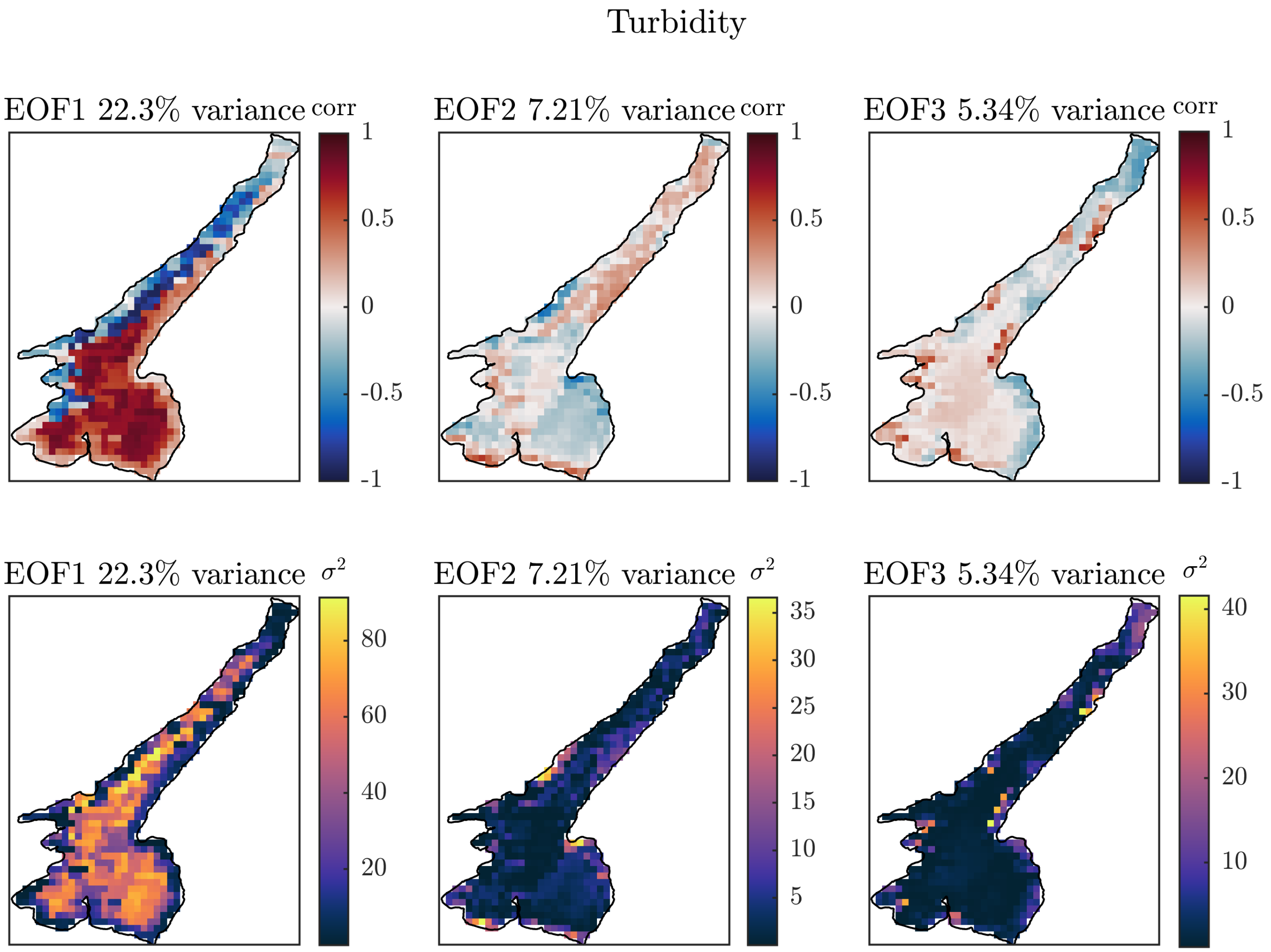

Figure 10. Correlation (top) and variance (bottom) associated to the three dominant EOFs of turbidity in Lake Garda.

Thermal profiles and climatology of mixed layer depth and Secchi depth from monitoring points.

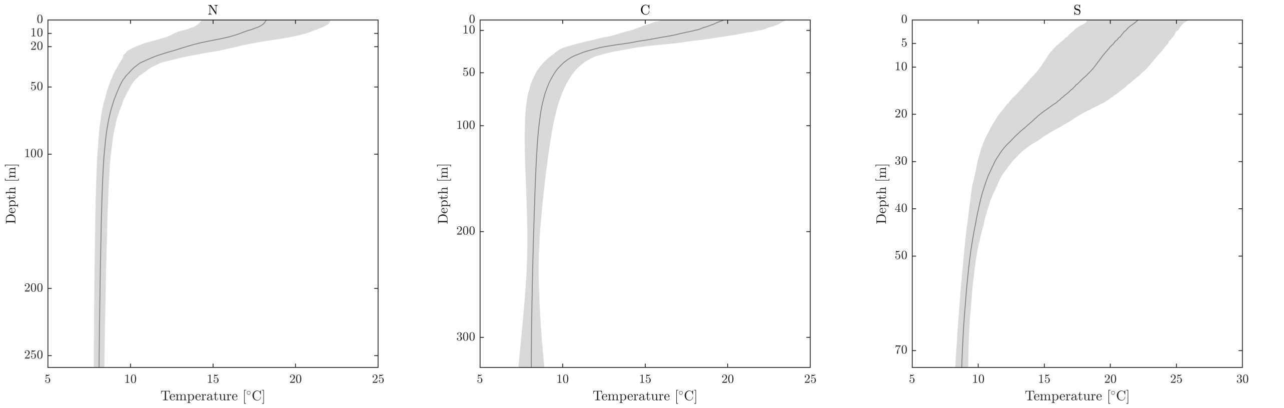

Figure 11. Water temperature profiles at the three monitoring stations during the stratified months (from May to September). Shaded grey area shows the standard deviation and black line the average over the time period 2002-2020.

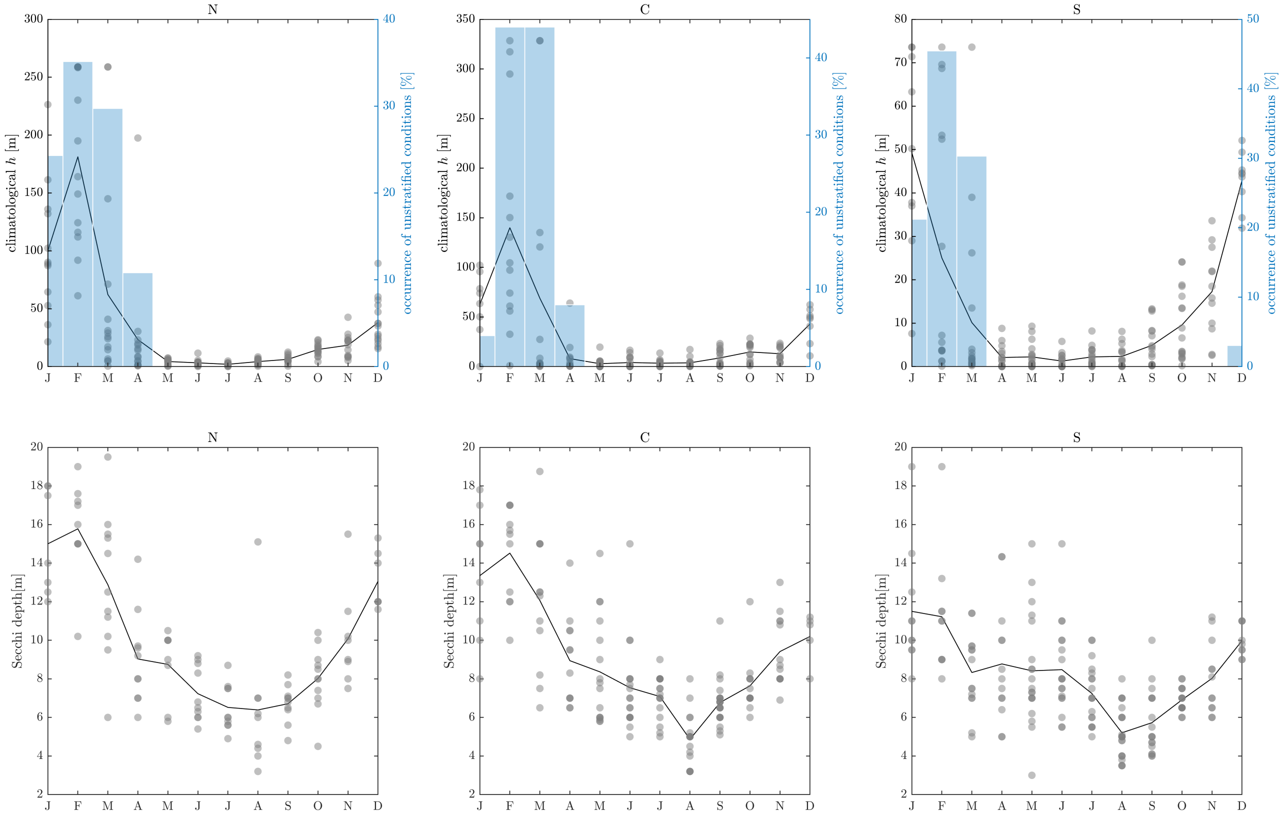

Figure 12. Climatology (black line: mean year average; dots: single values) of mixed layer depth (top plots) and Secchi depth (bottom plots) at monitoring stations (N, C, S, columns). Blue bars in top plots show the frequency of occurrence of unstratifed conditions in each month of the year. Mixed layer depth is computed as the depth where water temperature is colder than surface water temperature by more than 0.1°C. Absence of stratification is defined when the maximum thermal gradient along the water column is lower than 1°C).

Uncertainty associated to mixed layer depth estimation from LSWT anomaly

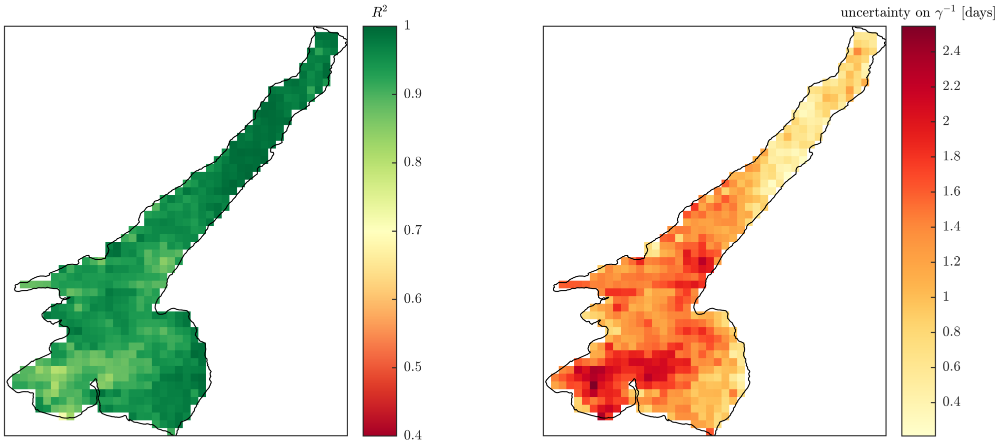

Figure 13. R^2 obtained from fitting eq. 9 (in main text) to the autocorrelation function of LSWT anomaly in each pixel of the maps and relative uncertainty associated to the estimated γ^{-1} time scale.

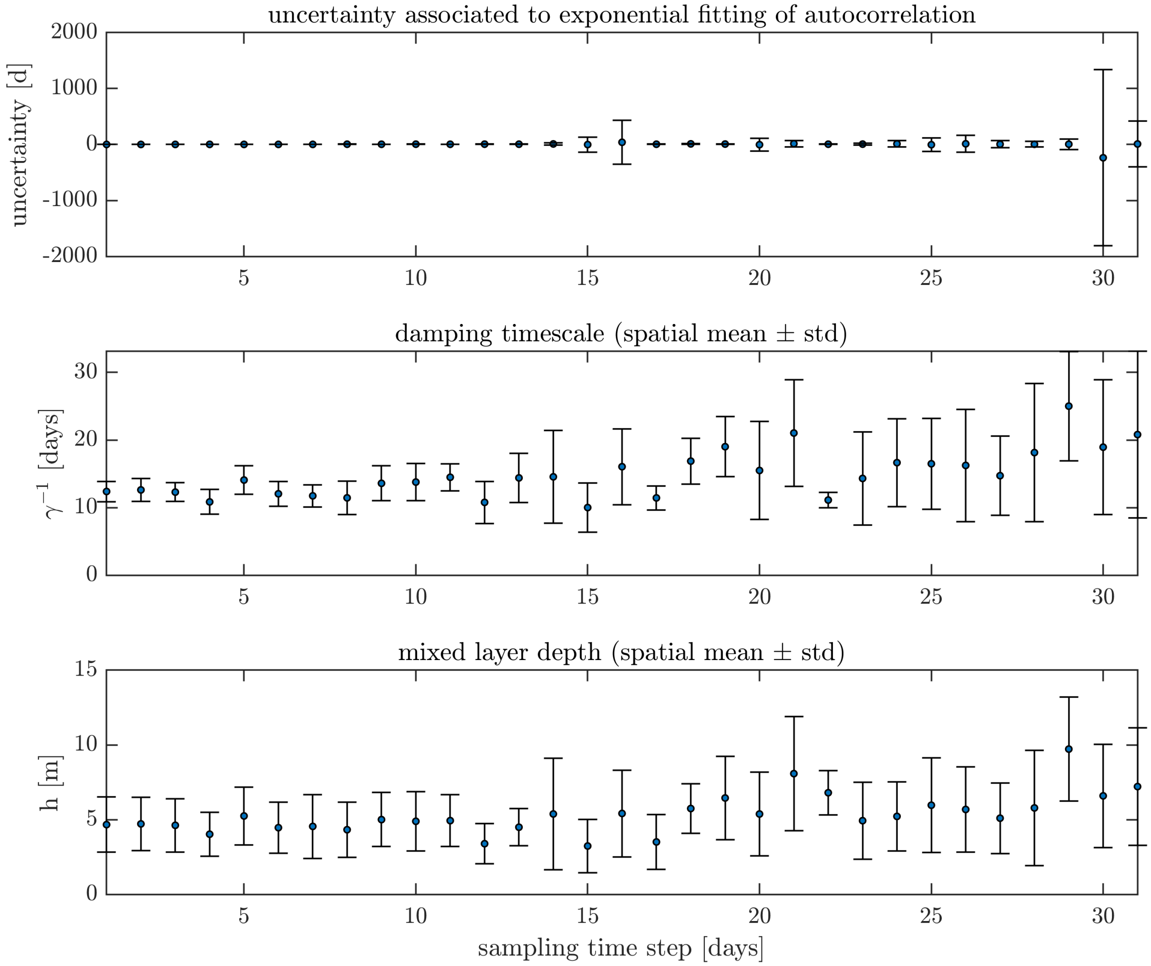

Figure 14. Uncertainty in the estimation of the damping timescale and mixed layer depth as a function of the sampling time step of LSWT anomaly in days.
